# Quality improvement intervention for school-based support teams for adolescents’ mental health in selected secondary schools in Gauteng: A study protocol

**DOI:** 10.1371/journal.pone.0353877

**Published:** 2026-07-16

**Authors:** Xolelwa Dyani, Olanrewaju Oladimeji, Moreoagae Bertha Randa

**Affiliations:** 1 Department of Public Health, Sefako Makgatho Health Sciences University, Faculty of Health Care Sciences, Pretoria, South Africa; 2 SAMRC/SMU Public Health Interventions, Innovations and Implementation Research Unit, Sefako Makgatho Health Sciences University, Pretoria, South Africa; Jawaharlal Institute of Postgraduate Medical Education and Research, INDIA

## Abstract

The prevalence of mental health disorders among adolescents poses a significant global health challenge, with approximately one in seven individuals aged 10–19 affected. The limited access to effective mental health services worsens this issue, particularly within schools, which are critical settings for early intervention. The study aims to develop and implement a school-based support team improvement intervention to enhance the mental health of adolescent learners in secondary schools. This study will employ a sequential explanatory mixed-methods approach design. The research will be conducted at selected secondary schools, and the participants for the study will be adolescents aged 13–19 and members of the school-based support team (SBST). Questionnaires will be administered to learners to determine the prevalence of common mental health challenges among adolescents and to determine the factors associated with mental health challenges among adolescents. A scoping review will also be conducted to examine existing interventions aimed at supporting learners with mental health challenges. Following the quantitative phase, interviews will be conducted with SBST members to explore the challenges and successes in supporting learners with mental health problems. The second phase will involve the development and implementation of the SBST quality-improvement intervention to support adolescents experiencing mental health challenges in secondary schools. The researcher will use Statistical Package for the Social Sciences (SPSS, 30.0.0) for quantitative data analysis and statistical interpretation. In addition, ANOVA will be employed to compare, identify, and understand specific factors that significantly impact adolescents’ mental health. The qualitative data will be analysed using Colaizzi’s seven-step framework and will be facilitated with NVIVO 15.

Ethical approval for this study was obtained from the Sefako Makgatho University Research Ethics Committee. Participation will be voluntary, and confidentiality will be maintained throughout data collection and analysis. The findings will be disseminated through international academic journals, conferences, and to school stakeholders.

## Introduction

The prevalence of mental health disorders among adolescents represents a significant global health challenge, with a majority of cases manifesting during this critical developmental stage [1]. Adolescence is characterized by rapid physiological, emotional, and social changes, which render individuals particularly vulnerable to various mental health challenges [2]. Research indicates that approximately one in seven young people aged 10–19 experience a mental health issue, contributing to 15% of the global burden of disease in adolescents [3]. The escalating incidence of mental health problems within this demographic poses dire implications, as early-onset conditions such as depression are frequently associated with adverse outcomes that persist in adulthood [3].

In South Africa, the burden of mental health issues among adolescents is increasingly recognized as a public health concern that affects a considerable proportion of the adolescent population [4]. Adolescents experience numerous stressors, including academic pressure, peer influences, unmet emotional needs, and the complexities of identity development [5]. [3]External factors, such as food and housing insecurity, family conflicts, gender-based violence, substance abuse, parental divorce, child-headed households, exposure to trauma, bullying, and financial difficulties, can significantly impact adolescents’ mental health and overall well-being, adversely affecting their academic performance.

The consequences of mental health issues among adolescents can be severe, leading to violence, harm, suicide, substance abuse, and poor academic performance [6]. Research also indicates a strong link between mental health problems and high dropout rates or absenteeism in schools [7]. These challenges can result in ongoing difficulties in education, employment, and social relationships later in life [8]. This underscores the importance of recognizing and addressing mental health issues early to prevent them from worsening into adulthood

A critical factor contributing to the global burden of adolescent mental health disorders is the limited access to affordable and effective mental health services, particularly in low- and middle-income countries like South Africa [9]. This situation is intensified by high rates of exposure to violence, trauma, and substance abuse among adolescents, which are associated with an increased likelihood of developing mental health issues [10]. Research conducted by [11]underscores the necessity of identifying effective and cost-efficient methods to address mental health challenges in low- and middle-income countries. Schools have been identified as optimal settings for early intervention, given that adolescents spend a substantial amount of their time in educational environments, and a study by [12]found that teacher-led interventions hold promise in mitigating the mental health challenges faced by students. [13]report that 98% of children aged 7–17 attend school, thus providing a vital opportunity for mental health support.

In response to the mental health needs of adolescents, the South African government has implemented several policies and interventions, such as the Integrated School Health Policy (ISHP), the Screening, Identification, Assessment, and Support policy (SIAS), and the District-Based Support Team. Despite these initiatives, schools face significant challenges in implementation, often due to institutional isolation, non-compliance, and inadequate resources [14]. The shortage of mental health professionals, including psychologists, nurses, and social workers, further hampers the delivery of mental health services in educational settings [15]. The inconsistency of support, coupled with the inequitable distribution of resources among schools, adversely affects the identification and support of learners facing mental health challenges [16].

At the school level, many schools in South Africa have established school-based support teams (SBSTs) to address the complexities faced by learners and educators. Teachers are crucial members of the SBST and play an essential role in recognizing learners who may be experiencing mental health issues because of their daily interactions with learners [14]. The SBSTs are recognized as pivotal structures within schools that provide support to learners with diverse needs, particularly regarding mental health [17]. However, research indicates that educators who are the main stakeholders of SBST regularly report feeling overburdened, underprepared, and lacking effective strategies to support students’ mental health [18].

### Aim

This study aims to develop and implement a quality improvement intervention to strengthen the capacity of SBSTs in helping adolescents with mental health challenges in selected secondary schools in Gauteng Province. The overall objectives of the study are four and are divided into two phases, as shown in Table 1 below.

**Table 1:** Objectives of the study.

Phase	Objectives
**Phase 1**	• To determine the prevalence and associated factors of mental health problems among adolescent learners in secondary schools. • To synthesize existing evidence on effective school-based mental health interventions. • To explore SBST experiences, challenges, and best practices in supporting learners with mental health challenges.
**Phase 2**	• To co-develop a quality improvement intervention for SBSTs. • To pilot a quality improvement intervention for SBSTs.

## Methods

### Research design

This study will follow a sequential explanatory mixed-methods research design. The quantitative data will be gathered and analyzed first to gain an understanding of common mental health challenges and associated factors among adolescents. This will be followed by the collection and analysis of qualitative data to explore the challenges and successes of SBST in supporting learners with mental health challenges.

During the intervention development phase, findings from the scoping review will be integrated with quantitative data on the prevalence and associated factors of mental health problems, as well as qualitative findings on stakeholders’ experiences and perspectives. The triangulation of evidence from these three sources will inform the development of a contextually relevant and evidence-informed intervention to promote adolescent mental health in secondary schools.

### Overview of the research approach

Phase 1: This phase will involve administering questionnaires to determine the prevalence and factors associated with common mental health challenges among adolescent learners. For the 2^nd^ objective, a scoping review will be conducted to synthesize existing evidence on interventions aimed at helping learners with mental health challenges, including SBST. The third objective will involve in-depth interviews with SBSTs to explore their challenges and successes in supporting adolescent learners with mental health challenges.

Phase 2: The development, pilot, and evaluation of quality improvement intervention. This phase will use a quantitative pre-post quasi-experimental design, and data will be collected from a group of adolescents before and after the implementation of the intervention to determine its effectiveness.

### Methods and design for phase 1 objective 1

#### Study design, setting, and population.

A descriptive quantitative cross-sectional survey will be conducted. The research will be conducted at selected secondary schools in Gauteng. Four secondary schools will be purposively selected; two in urban and two in township settings to ensure socioeconomic and demographic diversity. The first population will be adolescents aged 13–19 enrolled in grades 8–12. This specific age range is methodologically justified as it directly aligns with the typical student demographic found within secondary education (grades 8–12). making them the target population for this study.

#### Sampling, sampling size, and recruitment.

Stratified random sampling will be used to select the participants. Learners will be categorized according to their grade level and age. The estimated number of learners from the four selected schools is 6042. The total sample size is 440, which was calculated using Slovin`s formula. Class registers for Grades 8–12 will be used to identify all eligible learners. Within each grade stratum, learners will be assigned unique identification numbers. Approximately 15 learners per grade will then be selected using simple random sampling. The selection will be done by placing the numbered slips in a container, thoroughly mixing them, and drawing slips without replacement until the required sample size per grade is reached, thereby ensuring that each learner has an equal chance of being selected. The researcher will then explain the study to the selected learners, and prospective participants who express interest in the study will be provided with the necessary documentation, including informed consent forms for parents/guardians, assent forms for the adolescents, and detailed information leaflets. These materials are intended to be taken home to facilitate a comprehensive discussion with their parents or legal guardians regarding their potential involvement in the research. Parents will be requested to personally submit the signed consent forms at the school to ensure that students do not sign for themselves.

#### Inclusion and exclusion criteria.

The study population will include adolescent learners enrolled in grades 8–12 and who are between the ages of 13 and 19 and have signed parental/guardian consent and participant assent forms. On the other hand, any learner outside the defined age range of 13–19 years of age, who lacks both the required signed parental/guardian informed consent and participant assent forms, will be excluded from participation.

#### Data collection tools and methods.

The Patient Health Questionnaire-Adolescent (PHQ-A), Generalized Anxiety Disorder 7 (GAD 7), International Trauma Questionnaire – Child and Adolescent Version (ITQ-CA) and the Pediatric Adverse Childhood Experiences and Related Life Events Screener (PEARLS) are screening tools that will be used in this study to determine the prevalence and associated factors of mental health problems among adolescent learners in secondary schools.

The PHQ-A is a self-report questionnaire used to assess symptoms of depression in adolescents. It is a widely used tool in the field of mental health and helps professionals identify and monitor potential cases of depression in adolescents [19]. The GAD-7 questionnaire is a commonly used screening tool for assessing generalized anxiety disorder. It consists of seven questions scored on a scale from 0 to 3, and the total scores range from 0 to 21 [20]. The International Trauma Questionnaire – Children and Adolescent Version is a 22-item self-report measure focusing on the core features of Post Traumatic Stress Disorder and Complex PTSD in children and adolescents [21]. The Pediatric Adverse Childhood Experiences and Related Life Events Screener assesses adverse childhood experiences and other traumatic life events in pediatric patients [22]. The items in the tool include the Adverse Childhood Experiences categories and related life events, such as exposure to discrimination, food insecurity, housing instability, community violence, physical illness or disability of a caregiver, death of a caregiver, and forced separation from a caregiver.

#### Reliability, validity, and field testing of the instruments.

The selected tools have been validated and demonstrate reliability for screening adolescent mental health problems. Additionally, the tools will be pretested with 20 selected adolescents aged 13–19 years from two secondary schools that will not be included in the main study. 10 participants will be recruited from a public school located in a township, and a further 10 from a public school in the suburbs, representing different grades and age groups. This process will enable the researcher to identify and address potential issues with the instruments, thus enhancing the overall quality and accuracy of the study results.

#### Data analysis.

Quantitative data will be analyzed using SPSS version 30.0. Descriptive statistics analysis will be utilized to summarize the prevalence rates of different mental health conditions and demographic characteristics. Inferential statistics, including t-tests and chi-square tests, will be used to compare the prevalence of mental health issues across different demographic groups. Descriptive statistics for each survey item will be computed and summarized in the text, and the results will also be presented in tabular form for easy interpretation. Furthermore, frequency analysis will be performed to determine the valid percentages for responses to all survey questions, providing a comprehensive understanding of the participants’ perspectives.

The researcher will use a multivariate regression analysis as the statistical method to analyze factors associated with mental health problems. This method has been chosen due to its ability to simultaneously assess the impact of multiple independent variables on a dependent variable. Given the complex nature of factors contributing to adolescent mental health problems, the multivariate regression analysis will allow the researcher to assess associations between sociodemographic and psychological variables. This method can also help the researcher control potential factors that could influence the results, giving a better understanding of what affects adolescents’ mental health. In addition, ANOVA will be employed to identify and understand specific factors that significantly impact adolescents’ mental health, contributing to a more comprehensive analysis of the complex interplay among variables.

An experienced statistician will be actively involved in the analysis process to ensure the accuracy and reliability of the results. This expertise will strengthen the robustness of the data analysis and interpretation, ultimately leading to meaningful insights into adolescent mental health issues.

The primary outcomes of the study will be depressive symptoms, anxiety symptoms, and trauma-related symptoms, as measured by the PHQ-A, GAD-7, and ITQ-CA, respectively. Potential clustering of responses within schools and grades will be accounted for during analysis using appropriate statistical techniques, e.g., mixed-effects regression models or cluster-robust standard errors. Multivariable regression models will be used to identify factors associated with mental health outcomes while controlling for potential confounding variables, including age, sex, grade, school location, and adverse childhood experiences. Missing data will be assessed in terms of extent and patterns prior to analysis, and appropriate methods such as complete case analysis and the extent of the missing data will be applied. To reduce the risk of Type I error associated with multiple statistical comparisons, results will be interpreted using confidence intervals and effect sizes alongside p-values, with statistical significance set at p < 0.05.

### Methods and design for phase 1, objective 2

Objective 2 is to synthesize and review the existing literature on school-based support team interventions for learners with mental health challenges. A scoping review structure put forth by [23]will serve as the basis for the review: The review will adhere to a five-step process: (1) identifying the research topic; (2) locating relevant studies; (3) selecting studies; (4) charting the data; (5) compiling, summarizing, and reporting the findings. The last stage (consultation process) will be omitted because the review aims to synthesize existing literature rather than generate new insights or recommendations. Selection of articles will be detailed using the Preferred Reporting Items for Systematic Reviews and Meta-Analyses (PRISMA) guidelines. The researchers will create a thorough search plan by looking for articles published in English from 2015 to 2025. The researchers will conduct comprehensive search across several databases, including PubMed, Scopus, CINAHL, EBSCOhost, Google Scholar, OpenGrey, and the WHO Global Health Library. To identify relevant and potential grey literature, targeted searches will also be conducted on Theses. Two researchers will independently review and extract data to enhance reliability. In cases of disagreement, a third researcher will facilitate discussion and consensus. The selection process for the studies included in the review is scheduled to be completed within a 10-week timeframe. This process will strictly follow the comprehensive guidelines established in the PRISMA-ScR checklist.

### Methods and design for phase 1 objective 3

Objective 3 is to understand the challenges and successes of SBSTs in supporting learners with mental health challenges**.** For this objective, the researcher will adopt a qualitative approach using a descriptive phenomenological design. The goal of descriptive phenomenological research design is to understand how a group of people experienced a certain event and what it meant to them [24]. This method will be used as it will allow members of SBSTs to share their lived experiences, particularly their challenges and successes in supporting learners with mental health problems.

#### Population, sample, sample size, and recruitment strategy.

The second study population will be SBST members. The SBST is a formal structure that consists of selected teachers who are responsible for creating enabling, inclusive, and safe learning environments for all learners who experience barriers to learning [17]. The researcher will purposively sample 20 SBST members who have interacted with adolescents experiencing mental health challenges, with five members from each of the four selected schools. Data will be collected using an interview guide. Data collection and analysis will be conducted concurrently to determine the point at which data saturation is reached, defined as a stage at which no new themes emerge from the data. The proposed sample size of 20 SBST members is considered an initial estimate, as qualitative samples are guided by saturation rather than numbers. Should data saturation not be achieved within the initial sample, additional SBST members from other secondary schools with similar contextual characteristics will be recruited to ensure adequate thematic depth and completeness. The school principals will be informed about the study and asked for permission to access the SBST members before data collection commences. Participants will be provided with an overview of the study’s purpose and will be given an opportunity to ask questions for clarity. Informed consent will be sought for participation and for the use of audio tape from the participants. The interviews will be conducted in a location within the school chosen by each participant to enhance comfort. The researcher will conduct the interviews, and an experienced qualitative research assistant will take field notes to ensure consistency and capture non-verbal clues.

#### Inclusion criteria and exclusion criteria.

Members of the School-Based Support Team (SBST) who have at least two years of experience supporting learners with mental health challenges and are willing to participate and provide informed consent will be included. Those members who have never supported adolescent learners facing mental health issues will be excluded from participation.

#### Data analysis.

**Fig 1:**
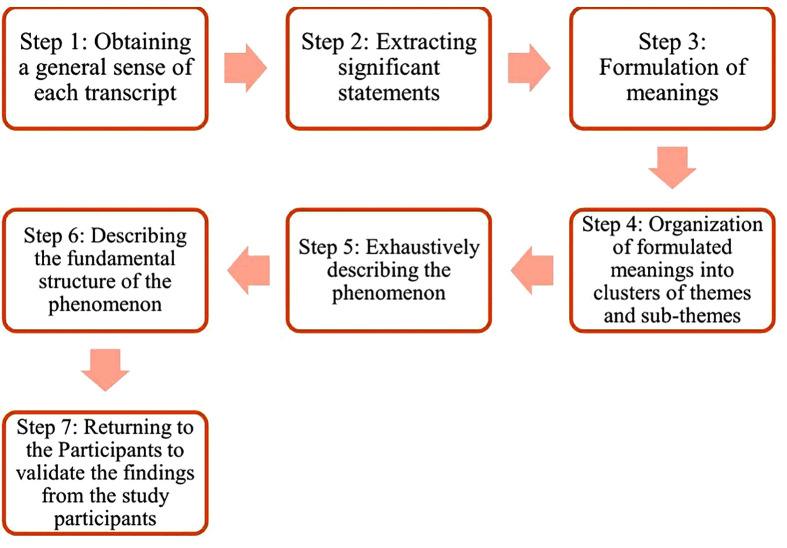
Colaizzi’s seven-step phenomenological framework.

Qualitative data will be analyzed using Colaizzi’s seven-step phenomenological framework [25]as shown in (Fig 1). In addition, data analysis will be facilitated with NVIVO 15.

#### Trustworthiness.

To ensure the trustworthiness of the study, four key criteria will be addressed: **credibility**, **transferability**, **confirmability**, and **dependability**. Participants will be given ample time to review the interview transcriptions to verify their contributions, allowing them to correct any inaccuracies. The researcher will provide a comprehensive description of the data collection methods, incorporating participants’ exact words to enhance transferability. The study will adhere strictly to its objectives, detailing the participant selection methods, sample size, and data collection procedures for potential replication. Data analysis will be conducted with the assistance of phenomenological experts, and the accuracy of transcriptions will be confirmed by comparing recordings to the transcripts. Confirmability will also be enhanced using NVivo 15 software.

#### Bias.

The researcher will consciously set aside personal biases to gain a thorough understanding of participants’ experiences on mental health challenges. By selecting participants from diverse schools, the researcher aims to capture a broad range of viewpoints and minimize selection bias. Additionally, direct quotations from participants will be prioritized to limit personal interpretations and reduce the potential for response bias.

### Methods and design for phase 2, objective 4

The final phase will involve developing the SBST quality-improvement intervention to support adolescents experiencing mental health challenges in secondary schools. Phase 2 forms part of the PhD study and will be undertaken after completion of Phase 1 data collection and analysis. The findings from Phase 1 will inform the design and implementation of the intervention in Phase 2. The intervention will focus on strengthening early identification of mental health concerns, improving referral pathways, and enhancing psychosocial support for affected learners. It is expected to include capacity-building for SBST members, the development of screening and referral guidelines, and the implementation of structured mental health awareness and support activities within schools. The final content and structure of the intervention will be refined based on evidence generated from the scoping review, as well as the quantitative and qualitative findings.

#### Study design, setting, population.

This phase will employ a pre-post experimental design and will utilize the same study setting and population as in Phase 1. The study population will be adolescents aged 13–19 who took part in the pre-intervention questionnaire.

#### Sampling and sample size.

Convenience sampling will be utilized; the sample will comprise learners who completed the pre-intervention questionnaire and who are subsequently willing to participate in the post-intervention questionnaire. The sample size was calculated using Raosoft’ s sample size calculator, yielding an estimated target sample of 206 participants.

#### Data collection tools and methods.

To establish the effectiveness of the intervention, the same questionnaires utilized in phase 1 to determine the prevalence and associated factors of mental health problems among adolescent learners in secondary schools will be administered. These instruments will be distributed pre-intervention and again post-intervention to the participating learners. A statistically significant difference in scores on the identified mental health measures between the pre- and post-intervention assessments will serve as the primary indicator of the intervention’s effectiveness.

#### Reliability and validity.

A questionnaire will be administered to adolescent learners before the quality improvement intervention, and the same questionnaire will be administered to the same adolescent learners after the intervention to assess its effectiveness. Once an intervention has been developed, it will be pilot-tested in a small sample of adolescents (pre-post design) to assess feasibility and acceptability. If the results are positive, the intervention will be tested for effectiveness in a large sample using a pre-post experimental design.

#### Data analysis.

SPSS 30.0.0 will be used to analyze the data. Various statistical techniques will be employed to examine the data and uncover patterns, correlations, and trends in mental health challenges among adolescents after the quality improvement intervention. This analysis aims to provide comprehensive insights into the impact of intervention on adolescent mental health.

### Ethics and dissemination

Ethical clearance was granted by the Sefako Makgatho University Research Ethics Committee (SMUREC/H/25/2025: PG). Permission was also sought and granted by the Department of Education (Reference Number: 8/4/4/1/2) to conduct the study in the selected schools. In addition, the researcher will make arrangements with the school principals to gain access and permission to engage with the participants. Due to the sensitive nature of the study, mental health support services will be organized on-site and provided when a need arises, and data collection will stop if any participant experiences emotional distress. Mental health support will be provided by a mental health care professional sourced from the Department of Education referral pathway. The results will be disseminated to the selected school and will also be shared with the Department of Education and the Department of Health. The study findings will also be published in Department of Higher Education and Training-accredited journals. In addition, the researcher will present the study at local and international academic research conferences.

### Informed consent (including assent from the participants)

For participants under 18, parental consent will be obtained before engagement, ensuring that parents are informed about the study’s objectives, risks, and benefits, allowing them to make informed decisions. Additionally, assent will be sought from learners under 18, recognizing their autonomy and allowing them to express their willingness to participate.

Participants’ rights, privacy, consent, secrecy, informed consent, anonymity, and possible harm are all included in the ethical considerations for this study. Each participant will receive a request for their written consent, emphasizing that their involvement in the study is entirely voluntary and not coerced.

To ensure the utmost confidentiality, all results will be presented anonymously, safeguarding the privacy of the data collected. No names will be used, instead, codes or numbers will be used to maintain privacy (POPIA, 2013). Participants will be thoroughly informed of their right to withdraw from participating in the study at any stage without facing any repercussions or penalties. The researcher is committed to transparency, assuring that all relevant information regarding the study will be openly shared with participants. There will be no attempts to deceive or mislead them in any manner, thereby fostering an atmosphere of trust and ethical integrity throughout the research process. All the data, including transcripts, questionnaires, recordings, checklists, etc., will be stored in a locked office and password-protected computer/hard drive in the researcher’s office. The data will be stored for 15 years and then destroyed according to institutional policy.

### Timeline

Participant recruitment started in June 2025 and is expected to be completed by March 2026, with all data collection anticipated to be completed by April 2026. The study is anticipated to be completed in December 2026, and the results are anticipated to be available by January 2027.

## Discussion

The prevalence of mental health issues among adolescents has emerged as a significant global concern. Because adolescents spend a considerable amount of time in schools, this setting is arguably the most crucial point for the early recognition and effective intervention of mental health issues. The SBST is mandated to create an effective learner support system that encompasses various aspects of student welfare, including mental health support. Research illustrates that teachers who are key role players in the SBST frequently experience feelings of being overwhelmed, pressured, and underprepared, lacking the effective strategies to support children’s mental health [18]. Using the existing structure of the SBST is advantageous as the team has already established relationships and gained valuable insight into the learners’ specific needs. This feasibility, therefore, provides a sustained system for mental health support that can easily align with local, national, and international mental health policies and initiatives.

In conclusion, the development of a quality improvement intervention for SBSTs aimed at supporting adolescents’ mental health challenges may yield improved mental health outcomes, facilitate better access to appropriate care, and enhance overall well-being among learners facing mental health challenges. Additionally, schools may become better equipped to meet the mental health needs of their learners, potentially resulting in improved academic performance, decreased absenteeism, and a more positive educational environment for both learners and educators.
